# Balance Remains Impaired after Hip Arthroplasty: A Systematic Review and Best Evidence Synthesis

**DOI:** 10.3390/diagnostics12030684

**Published:** 2022-03-11

**Authors:** Giorgio Di Laura Frattura, Vittorio Bordoni, Pietro Feltri, Augusto Fusco, Christian Candrian, Giuseppe Filardo

**Affiliations:** 1 Service of Orthopaedics and Traumatology, Department of Surgery, EOC, 6900 Lugano, Switzerland; giorgio.dilaurafrattura@eoc.ch (G.D.L.F.); vittbordoni@live.com (V.B.); christian.candrian@eoc.ch (C.C.); ortho@gfilardo.com (G.F.); 2 Occupational and Environmental Medicine, Università degli Studi di Milano, 20122 Milano, Italy; 3 UOC Neuroriabilitazione ad Alta Intensità, Dipartimento di Scienze dell’Invecchiamento, Neurologiche, Ortopediche e della Testa-Collo, Fondazione Policlinico Universitario, A. Gemelli IRCCS L. go Francesco Vito n. 1, 00168 Roma, Italy; augusto.fusco@policlinicogemelli.it; 4 Faculty of Biomedical Sciences, Università della Svizzera Italiana, Via Buffi 13, 6900 Lugano, Switzerland

**Keywords:** balance, hip, arthroplasty, proprioception

## Abstract

Background: Hip arthroplasty (HA) is the most common intervention for joint replacement, but there is no consensus in the literature on the real influence of this procedure on balance, or on what factors in the pre-operative, surgical, and post-operative stages may affect it. Purpose: To synthesize the evidence on how Hip Arthroplasty (HA) affects balance, identifying pre-operative, surgical, and postoperative risk factors that may impair balance in HA patients, with the aim to improve patients’ management strategies. Methods: A literature search was performed on PubMed, PeDRO, and Cochrane Collaboration on 25 May 2021. Inclusion criteria: clinical report of any level of evidence; written in English; with no time limitation; about balance changes in hip osteoarthritis (OA) patients undergoing HA and related factors. Results: 27 papers (391 patients) were included. Overall, the evidence suggested that balance is impaired immediately after surgery and, 4–12 months after surgery, it becomes better than preoperatively, although without reaching the level of healthy subjects. A strong level of evidence was found for hip resurfacing resulting in better balance restoration than total HA (THA), and for strength and ROM exercises after surgery positively influencing balance. Conclusion: Both the surgical technique and the post-operative protocols are key factors influencing balance; thus, they should be carefully evaluated when managing hip OA in patients undergoing HA. Moreover, balance at 4–12 months after surgery is better than preoperatively, although without reaching the level of the healthy population. Attention should be paid in the early post-operative phase, when balance may be impaired in patients undergoing HA.

## 1. Introduction

Hip arthroplasty (HA) is the end-stage intervention for patients affected by hip osteoarthritis (OA). It is the most common joint replacement procedure and the number of patients undergoing prosthetic implantation is progressively growing due to the ageing population [1,2]. Even though HA shows excellent clinical results and has sometimes been referred to as “the operation of the century” [1], the risk of falls remains high, with the inherent detrimental consequences for the elderly and fragile patients [3]. Around one-third of the over 65 people living in the community are at risk of falling at least once a year, with consequent fractures or major injuries in 10% of falls [4]. Falls have been supposed to increase in the next years due to the ageing of the world population, and they are an important cause of death among the elderly [5]. Often, falls are associated with a loss of balance, which is a complex function regulated by the integration of the sensory inputs coming from the somatosensory, the visual, and the vestibular systems, as well as by the response capacity of muscles [6].

Since hip OA causes damage to the proprioceptors, the risk of loss of balance—and therefore of falling—is even higher in people affected by hip OA [7,8]. After HA, the patients’ balance is impaired by different factors: the muscles are weaker, the lever arms are changed, the operated leg can be shorter than the other one and it bears less weight, there is a global reduction in range of movement, and the surgical capsular excision can cause additional damage to the proprioceptors [9,10,11]. Nevertheless, there is no consensus on the real influence of HA on balance, or on what factors in the pre-operative, surgical, and post-operative stages may affect it. Thus, it is important to determine exactly how HA may influence balance, and to identify the factors that may lead to an increased risk of falls in HA patients.

The aim of this paper was to perform a systematic review on the influence of HA on the balance of hip OA patients, evaluating the actual balance of operated patients, and identifying which pre-operative, surgical, and postoperative factors associated with HA increase the risk of falling. Moreover, the impact of different surgical procedures was analysed.

## 2. Methods

### 2.1. Source of Data and Data Extraction

On the 25 May 2021, three medical electronic databases (PubMed, PeDRO, and Cochrane Collaboration) were scanned to find relevant papers, using the following terms: ((hip arthroplasty) OR (hip replacement)) AND ((proprioception) OR (joint position sense) OR (sensorimotor) OR (postural control) OR (balance) OR (balance control)). The Preferred Reporting Items for Systematic Reviews and Meta-analysis (PRISMA) guidelines were followed [12].

Papers were first screened by title and abstract. Inclusion criteria: clinical study of any level of evidence, written in English, with no time limitation, about how, and because of what factors, balance changes in hip OA patients undergoing HA. Exclusion criteria: languages other than English, non-clinical study, review, or case report. The full text of the selected articles was screened to verify that the inclusion criteria were met. Manual screening of the references of the selected papers was also performed. Relevant data (type of study, number of patients, demographics, follow-up length, balance assessment, factors influencing balance, correlation with clinical outcome, and influence of pre/post-rehabilitation programs) were extracted from each paper and collected in a single spreadsheet. Two authors (G.D.L.F. and V.B.) performed independently the screening process, the review of the papers selected, and the extraction and tabulation of the data; the data extracted were then compared and a consensus was reached. The main aspects analyzed, related to balance in patients undergoing HA, were treatment-related changes, pre-operative, surgical, and postoperative risk factors.

### 2.2. Risk of Bias Assessment and Best-Evidence Synthesis

The risk of bias was studied using the Cochrane Collaboration’s tool [13] modified by Eijgenraam et al. [14]: two reviewers (G.D.L.F. and V.B.) independently scored all the papers according to a list of eight questions: two questions concerning selection bias, four information bias, and two confounding bias. The two reviewers discussed their findings and, if needed, asked a third reviewer (G.F.) to reach a consensus. Low risk of bias was defined as “yes” having been answered to at least six questions, with at least one “yes” in each risk category (selection, information, and confounding bias). A moderate risk of bias was defined as ‘yes’ having been answered to at least five questions, with at least one ‘yes’ in two categories. All the other cases were considered as having a high risk of bias.

Finally, since a meta-analysis could not be performed due to a lack of homogeneity, a best-evidence synthesis was performed using the algorithm developed by van Tulder et al. [15] and Eijgenraam et al. The following ranking of evidence level was used:

- Strong evidence, if a result was reported by two or more studies with low risk of bias and with findings that were, overall, at least 75% consistent across studies.
- Moderate evidence, if a result was reported by one study with low risk of bias and by two or more studies with moderate/high risk of bias, or if it was reported by two or more studies with moderate/high risk of bias and with findings that were, overall, at least 75% consistent across papers.
- Limited evidence, if a result was reported by one or more studies with moderate/high risk of bias, or if it was reported by one study with low risk of bias study and with findings that were, overall, at least 75% consistent across papers.
- Conflicting evidence, with conflicting findings (<75% of the studies reporting consistent findings).

## 3. Results

### 3.1. Characteristics of the Included Studies

**Figure 1:**
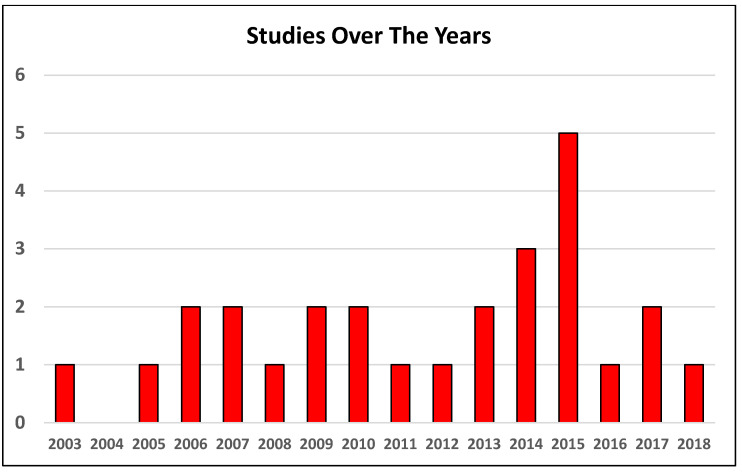
Temporal trend of the published papers about the influence of HA on balance.

**Figure 2:**
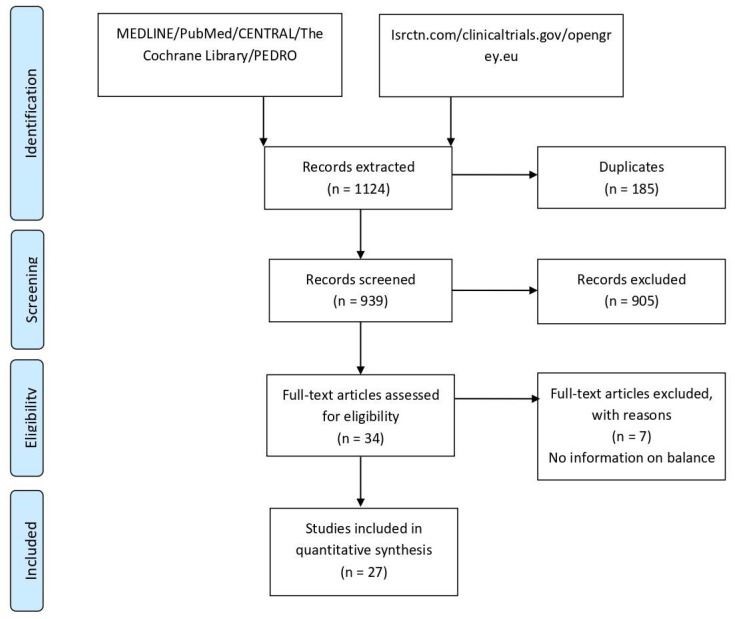
PRISMA of the retrieved studies.

The systematic review of the literature revealed that, in the last 10 years, the number of papers analyzing how HA influences balance has been increasing (Figure 1). The database search identified 939 articles. After screening and selecting abstracts according to inclusion/exclusion criteria, 34 full-text articles were assessed for eligibility. Of these, seven were excluded because they did not meet the inclusion criteria, leaving 27 papers (Figure 2) (four retrospective and 23 prospective) (4 of which were randomized controlled trials); the follow-up length ranged from 2 weeks to 10 years. The selected articles analyzed a population of 391 patients, with a female/male ratio ranging from 0.8 to 4.5, a mean age from 49 to 71 years, and a mean BMI from 23 to 31. The evaluation approaches were heterogeneous, with different studies using different clinical scales (Western Ontario and McMaster Universities Arthritis-WOMAC, Harris Hip, SF-36, EuroQol) and tests (Timed Up and Go, ABC Activities-specific Balance Confidence score, ROM, Berg Balance Score, Motor Control test, Romberg test, Merle d’Aubignè-postel test, gait analysis, force platform, 6- and 10-min Walking test, single and double leg standing). Of the 27 studies included, only two defined “balance”: one study defined it as the ability to stand on one leg for 10 s, and the other as trunk pitch and roll movement. Additional details in Supplementary Material Table S1.

### 3.2. Risk of Bias and Best-Evidence Assessment

The risk of bias and best-evidence synthesis have been investigated according to the following categories: treatment-related changes, preoperative, surgical, and post-operative risk factors.

#### 3.2.1. Treatment-Related Changes

**Figure 3:**
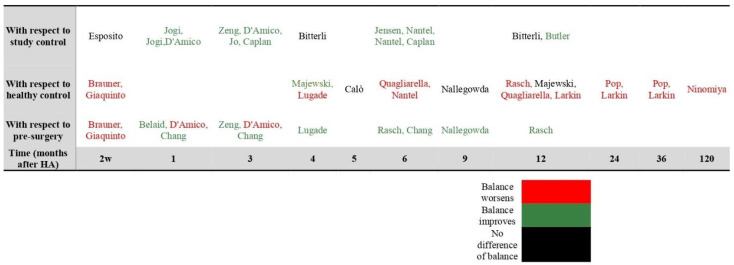
Change in Hip Balance.

In this case, 24 of the selected papers investigated treatment-related changes. Two studies reported that 2 weeks after surgery the balance of the patients was worse than at pre-op. One study reported that in the first month after surgery balance was worse than at pre-op, while two studies reported improvement. One study reported that 3 months after surgery balance was worse than at pre-op, while two studies reported improvement. Five studies reported that 4 to 12 months after surgery balance was better than at pre-op. Nine studies showed that 0.5, 1, 3, 4, 6, 12, 24, 36 and 120 months after surgery balance was worse than in healthy patients, while three studies found that 5, 9, and 12 months after surgery the balance of the patients was comparable to that of healthy subjects. Additional details in Table 1 and Table 2 and Figure 3.

#### 3.2.2. Pre-Operative Risk Factors

A limited level of evidence was found for the fact that neither BMI (Kiss [16,17]—limited risk of bias), Butler [16,17]—moderate risk of bias), gender (Pop [18]—limited risk of bias), nor sensory-motor exercises before surgery (Bitterli [19]—low risk of bias) influence balance.

#### 3.2.3. Surgical Risk Factors

A strong level of evidence was found for the fact that hip resurfacing is more effective than total hip arthroplasty at restoring balance (Nantel [20,21,22,23,24], Larkin [20,21,22,23,24], Caplan [20,21,22,23,24], and Jensen [20,21,22,23,24]—low risk of bias; Nantel—moderate risk of bias [20,21,22,23,24]), while a conflicting level of evidence was found regarding the influence of the type of surgical approach on balance, with no consensus among different authors on which approach provides the best outcome (Kiss [16,25,26], Holnapy [16,25,26], and Chang [16,25,26]—low risk of bias).

#### 3.2.4. Post-Operative Risk Factors

A strong level of evidence was found for the fact that strength and ROM exercises after surgery improved the balance of the patients (Nantel [27], Rasch [27], and Pop [27]—low risk of bias, Calò [27], Nantel [27], Brauner [27], Jogi [27], and Zeng [27]—moderate risk of bias). The exercises were performed for 2–12 weeks and their intensity ranged from a few minutes per day to 1 h per day. A moderate level of evidence was found for the fact that specific balance exercises improved the balance of the patients (Jogi [27] and Jogi [27]—moderate level of evidence). A limited level of evidence was found for the fact that the use of crutches (Esposito [27]: low risk of bias) or of a wedge under the foot (D’Amico [28]: low risk of bias) does not lead to improvements of balance.

## 4. Discussion

The main finding of this study is that the balance of patients with hip OA improves after HA, but it is not completely restored.

Since so many systems regulate balance, surgery—the main function of which is to restore the function of the target joint—is only partially able to completely restore balance when it is impaired as severely as it is by hip OA. Though HA does help restore balance, the extent to which it is effective in doing so varies in time. In particular, this review documented that up to 2 weeks after surgery the balance of HA patients is worse than it was before the operation. While balance is impaired immediately after surgery, more conflicting results have been reported for up to 3 months. Afterwards, the majority of the studies report that balance improves, and all the studies agree that at 4–12 months after surgery balance is better than at pre-op. However, despite this improvement, the majority of the retrieved studies state that the balance of HA patients is worse than that of healthy subjects. This finding is further strengthened by the recent work of John et al. [29], which found persisting asymmetries between the operated and the non-operated leg four to five years after THA. A persisting impairment of balance in patients who underwent HA might be explained by the fact that this type of surgery is not able to reverse the damage that OA causes to certain tissues (particularly the hip joint capsule and the soft tissues around it) which are rich in proprioceptors and therefore highly correlated with balance.

Balance is a topic of particular interest to researchers because it is highly correlated with the risk of falling, and falls, especially in the elderly, represent a serious risk of injury (25–60%, as reported by Kannus et al. [30]). Moreover, the number of falls is expected to increase over the next years, because the average age of the population keeps increasing. In patients affected by hip OA, the risk of falls is estimated to be 50%. After HA, the risk remains high, with 36% of people falling at least once in the first year after surgery [31,32]. Ikutomo et al. [32] analyzed patients living independently who had undergone HA and found that the most common causes of falls were tripping and loss of balance. In addition, it was found that these patients had a lower physical function and worse gait characteristics than people who never fell. In another study, the same authors documented that the presence of gait abnormalities is a useful screening tool to predict falls in a patient affected by hip OA who had undergone THA [33]. Since the reduction of balance in the early period following HA likely leads to falls, an improvement in patient management in this period should be pursued to mitigate the risk of falling [34].

Pre-operative, surgical, and postoperative risk factors were analyzed, and the quality of the evidence gathered was evaluated by performing a best-evidence synthesis. Strong evidence was found for the fact that the type of surgery performed influences how much balance improves. In particular, it was found that surface hip arthroplasty is better than total hip arthroplasty at restoring balance. The less invasive intervention, surface hip arthroplasty, likely causes less damage to the tissues (and therefore to the proprioceptors), consequently leading to a lower impairment of balance [20,21,22,23,24]. Surface hip arthroplasty is also more effective at restoring the correct biomechanics of the hips, since it leads to a more precise reconstruction, in which the hip center of rotation is preserved, thus not modifying the lever arm of the abductor muscle [35]. However, this conclusion is not shared by all authors [36]. In addition, the femoral head of the prosthesis is similar to the native in size and shape, which means that the loading distribution will be closer to physiological conditions than it would if total hip arthroplasty was performed [37]. Unfortunately, surface hip arthroplasty has also some disadvantages such as the risk of femoral neck fractures (associated with the surgeon’s learning curve) [38] and of acetabular and femoral component loosening (although quite infrequent) [39]. Moreover, literature reports that metal-on-metal implants are not indicated for patients with chronic kidney disease because of the risk of metallosis [40].

Strong evidence also exists supporting that post-operative training programs improve balance after HA. Different authors analyzed the effects of strength and ROM exercises after surgery. All the authors reported better early functional and balance outcomes compared to those patients who did not perform post-surgery exercises [18,20,21,41,42,43]. Unfortunately, no comparison between different training timing has been performed, since no study reported it.

A moderate level of evidence was found for the beneficial effect of specific balance training following surgery [44,45]. These results are particularly important, as they indicate that balance can be restored effectively even in the early period after surgery, which is when the risk of falling is at its highest. However, one year after the intervention, no differences from the control group were found. Further research efforts should be devoted to investigating how to prolong the positive effects of specific training on balance after the early period following the surgery, in particular by defining which are the most effective post-operative protocols. Training programs typically involve static and dynamic exercises to strengthen the muscles, the proprioception, and the overall balance of the patients, to attain optimal weight distribution symmetry. Unfortunately, while most rehabilitative protocols include a combination of these elements, there is no universal, standard protocol that is superior to the others, thus being used in all situations and for all patients. This leads to the use of many different protocols, making it difficult to determine if post-operative rehabilitation is an effective tool in restoring balance.

Pre-operative factors that may hinder balance recovery were also analyzed. A limited level of evidence was found for the fact that gender, BMI, and sensory-motor training before surgery does not influence balance recovery. Since HA causes impairment of sensory-motor function, sensory-motor training has been proposed as a rehabilitative treatment for a patient undergoing HA. However, the available literature does indicate that this type of treatment is effective at restoring balance. The observed lack of influence of pre-operative training on post-operative balance might be explained by the fact that balance is regulated by systems (i.e., the sensorimotor, visual, and vestibular systems) that might not benefit from the training. Another explanation could be that HA causes damage to the hip joint capsule and the nearby soft tissues rich in proprioceptors, damage that no rehabilitation can reverse. In this regard, different surgical approaches might affect the soft tissues differently. However, conflicting evidence was found regarding the effectiveness of different surgical procedures: anterolateral and posterior approaches were compared since they affect different anatomical structures around the hip. Kiss et al. [16] found that the anterolateral approach leads to better clinical outcomes and to better pelvis motion and gait analysis normalization in the patients they studied, and this finding is reported to be caused by the fact that, in this approach, the gluteus medius and the posterior capsule remain intact, thus enabling the compensatory mechanisms of the pelvis. On the contrary, Holnapy et al. [25] found that the posterior approach was superior at improving balance after THA, suggesting that this approach better preserves the joint capsule, thus sparing more proprioceptors. The type of surgical approach performed influences several aspects related to the clinical outcome other than the restoration of balance, such as the number of complications, the speed of recovery, and the outcome. Nevertheless, there is a need, which should be addressed by further studies, to explain the impact of different surgical procedures on restoring balance, to gain a more complete understanding of the benefits and drawbacks of the type of HA performed. Moreover, it would be interesting for future studies to address the gender differences in restored balance since males and females have different anatomy and degrees of degeneration in somatosensory function [46].

The main limitation of this study is that because the literature presents heterogeneous evaluation approaches, with several clinical scales and tests, it was unfeasible to evaluate aspects related to balance. “Balance” itself is poorly defined, and indeed many authors do not even define the term. The intrinsic complexity of balance leads to heterogeneous definitions, as well as to heterogeneous methods to measure it and attempt to improve it, making a synthesis of the results reported by the literature in this field challenging. 

A second limitation of this study is the small number of patients analyzed (391) and the low number of RCTs (4) included. Nevertheless, the studies available made it possible to perform a best-evidence synthesis and to underline interesting findings both on balance changes and on pre-operative, surgical, and postoperative factors that can play a role in restoring balance after HA.

In conclusion, though the literature supports the importance to address balance, further studies are needed to identify specific risk factors, and to determine what are the most suitable pre-operative, surgical, and post-operative aspects for the development of protocols to properly manage patients, improving balance and reducing the risk of falls and their dangerous consequences in the fragile elderly population of hip OA patients undergoing hip replacement.

The systematic review revealed a growing interest in balance changes in patients undergoing HA. Though balance in hip OA patients improves for some months after HA, it is never completely restored. In particular, there is a significant impairment of balance in the early period (2 weeks) after surgery. In this phase, attention should be paid to properly manage and protect patients undergoing HA. Afterwards, balance becomes better than pre-op at 4–12 months after surgery, although it never reaches the level of the healthy population. The best-evidence synthesis performed in this paper identified factors able to influence balance: a strong level of evidence was found for hip resurfacing being superior to THA, and for strength and ROM exercises after surgery being beneficial; a moderate level of evidence for the effectiveness of specific balance exercises. A limited or controversial level of evidence was found for other factors, which prompts further research to identify factors and specific protocols that should be considered to improve balance in hip OA patients undergoing HA.

## Figures and Tables

**Table 1:** Treatment-related changes.

Predictor	Significant Association (Level of Evidence)	No Significant Association	Level of Evidence
Exercise before THA	Bitterli (L)		limited
Strength and ROM exercise post THA	Nantel (L), Calò (M), Nantel (M), Rasch (L), Brauner (M), Joji (M), Joji (M), Zeng (M), Pop (L)		strong
BMI		Kiss (L), Butler (M)	limited
Balance exercise post surgery	Joji (M), Joji (M)		moderate
Set-up crutch	Esposito (L)		limited
Surgical approach	Kiss(L) Holnapy (L) Chang (L)		conflictual
Female		Pop (L)	limited
Type of intervention	Natel (M), Natel (L), Larkin (L), Caplan (L), Jensen (L)		strong
Proprioception	Nallegowda (M), Jo (M), Larkin (L)		conflictual

**Table 2:** Risk of Bias of the included studies.

Author	Year	Risk of Bias
Nallegowda	2003	moderate
Majewski	2005	low
D’Amico	2006	moderate
Giaquinto	2006	moderate
Belaid	2007	moderate
Nantel	2007	low
Lugade	2008	moderate
Calò	2009	low
Nantel	2009	moderate
Quagliarella	2010	low
Rasch	2010	low
Bitterli	2011	low
Kiss	2012	low
Holnapy	2013	low
Larkin	2013	low
Brauner	2014	moderate
Caplan	2014	low
Jogi	2014	moderate
Butler	2015	moderate
Chang	2015	low
Jensen	2015	low
Jogi	2015	moderate
Zeng	2015	moderate
Jo	2016	moderate
Esposito	2017	moderate
Ninomiya	2017	low
Pop	2018	low

## Supplementary Materials

The following supporting information can be downloaded at: https://www.mdpi.com/article/10.3390/diagnostics12030684/s1, Table S1: Details of the included studies.
