# Supplementary material for: Balance Remains Impaired after Hip Arthroplasty: A Systematic Review and Best Evidence Synthesis
Source: Diagnostics (Basel). 2022 Mar 11;12(3):684. doi: 10.3390/diagnostics12030684 (PMC8946928; doi:10.3390/diagnostics12030684)
Supplement: Supplementary file 1 [file diagnostics-12-00684-s001.zip › diagnostics-1603060-supplementary.pdf]

Supplementary Material Table S1 Details of the included studies

Author	*Patients (f-m)-age-BMI*	*Study design*	*Follow-up (months)*	*Balance definition*	*Risk factors*	*Measurement*	*Comparison*
Nallegowda 2003	30(nr-nr)-nr-nr	prospective	9	no	nr	dynamic posturography	pre-post surgery. Healthy control
Majewski 2005	25(11-14)-67-nr	prospective	12-4 months	Yes: trunk pitch and roll movement	nr	gait,mTUG, stance stand	pre-post surgery. Healthy control
D'Amico 2006	90(nr-nr)-66.5-nr	prospective	nr	no	nr	nr	pre-post surgery. Sperimental control
Giaquinto 2006	20(14-6)-70.2-nr	prospective	2weeks	no	nr	gait in the water	pre-post surgery
Belaid 2007	10(4-6)-69.8-nr	prospective	2weeks -1	no	nr	platf (CoP), TUG, lateral reach,test, musc strength, 10MWT,FIM,VAS	pre-post surgery. Leg healthy
Nantel 2007	20(9-11)-49-30	prospective	6	no	hip resurfacing-THA. 6-8weeks training	postural tasks	THA controls. Healthy control
Lugade 2008	20(6-14)-57-31.9	prospective	1day-6weeks -16w	no	nr	gait, Harris hip, force platf	pre-post surgery, healthy matched control
Calò 2009	23(13-10)-64.3-nr	prospective	4	no	4weeks rehabilitation	SOT, MCT, AT	Healthy control
Nantel 2009	10(5-5)-51-28.5	prospective	1day-6	no	hip resurfacing-THA.6-8weeks training	10MWT, Kynetic analysis, muscle strenght, WOMAC, Merlè d'Aubignè-postel	THA control. Healthy control
Quagliarella 2010	81(nr-nr)-64.1-nr	retrospective	12-6 months	no	nr	platform	Healthy control

Rasch 2010	22(18-4)-67-nr	prospective	24-6 months	no	10weeks training post-op	muscle strength, balance (platf),gait	pre-post surgery
Bitterli 2011	39(19-22)-65.4-nr	prospective RCT	10day-4-12	no	4w pre-op sensorimotor training	SF-36,WOMAC,BBS (platf)	pre-post surgery. Control no training
Kiss 2012	80(47-33)-71.3-nr	prospective	2003/6/12	no	direct lateral or anterolateral, BMI	HHS, gait,	healthy control
Holnapy 2013	45(36-9)-nr-nr	prospective	6weeks-3-6	no	direct lateral or anterolateral or posterior	perturbation platform	pre-post surgery.-other approaches
Larkin 2013	100(38-62)-nr-nr	prospective	nr	nr	hip resurfacing and THA	UCLA score, Harris hip, dynamic postural stability	pre-post surgery, healthy matched control, different intervention
Brauner 2014	61(27-34)-61.8-nr	prospective	2weeks	no	2weeks rehabilitation post-op	interlimb vertical force and dynamic stab during squat	healthy control
Caplan 2014	14(1-14)-63.8-nr	prospective	12-3 months	no	hip resurfacing-THA	TUG, vertical ground reaction force	THA control. Healthy control
Jogi 2014	26(nr-nr)-64-nr	prospective RCT	1 1/2	no	5weeks training e balance train post-op	Berg Balance, TUG, WOMAC, ABC test	no training control
Butler 2015	75(nr-nr)-66.1-31	retrospective	12	yes	BMI	unilateral stand position	control joints replacement
Chang 2015	23(13-10)-60-nr	prospective	2w-6w-3-6	no	minimally invasive THA	Vas, Berg balance, FIM,A BC, foot placement	pre-post surgery
Jensen 2015	38(13-25)-55-27.9	prospective RCT	6-2 months	no	hip resurfacing-THA	gait deviation index	pre-post surgery. THA control. Leg healthy

Jogi 2015	63(41-22)-67-nr	prospective RCT	6weeks	no	5weeks training e balance train post-op	platform (ellipse area)	no training control
Zeng 2015	59(28-31)-64-26.7	prospective random	6-3 months	no	12weeks training post-op	WOMAC,6MWT,TUG,UPST, ROM	no training control
Jo 2016	31(11-20)-65.4-nr	retrospective	3	no	nr	Berg balance, JPS, QOL, sf-36. (Proprioception) muscle mass	pre-post surgery
Esposito 2017	30(nr-nr)-69-28	prospective randomized	3days	no	crutches set-up post-op	VAS, Oxford hip score, platform	set up group
Ninomiya 2017	58(48-10)-68-22	retrospective	120	no	nr	muscle strength, single limb stance time, MWS, fall rate, IPAQ	healthy control
Pop 2018	55(31-24)-56-nr	prospective	24-36	no	4weeks rehabilitation. Female	platform (stabilometric), romberg	healthy control

**Author**	**Patients (f-m)-age-BMI**	**Study design**	**Follow-up (months)**	**Balance definition**	**Risk factors**	**Measurement**	**Comparison**
Nallegowda 2003	30(nr-nr)-nr-nr	prospective	9	no	nr	dynamic posturography	pre-post surgery. Healthy control
Majewski 2005	25(11-14)-67-nr	prospective	12-4 months	Yes: trunk pitch and roll movement	nr	gait,mTUG, stance stand	pre-post surgery. Healthy control
D'Amico 2006	90(nr-nr)-66.5-nr	prospective	nr	no	nr	nr	pre-post surgery. Sperimental control
Giaquinto 2006	20(14-6)-70.2-nr	prospective	2weeks	no	nr	gait in the water	pre-post surgery

Belaid 2007	10(4-6)-69.8-nr	prospective	2weeks -1	no	nr	platf (CoP), TUG, lateral reach,test, musc strength, 10MWT,FIM,VAS	pre-post surgery. Leg healthy
Nantel 2007	20(9-11)-49-30	prospective	6	no	hip resurfacing-THA. 6-8weeks training	postural tasks	THA controls. Healthy control
Lugade 2008	20(6-14)-57-31.9	prospective	1day-6weeks -16w	no	nr	gait, Harris hip, force platf	pre-post surgery, healthy matched control
Calò 2009	23(13-10)-64.3-nr	prospective	4	no	4weeks rehabilitation	SOT, MCT, AT	Healthy control
Nantel 2009	10(5-5)-51-28.5	prospective	1day-6	no	hip resurfacing-THA.6-8weeks training	10MWT, Kynetic analysis, muscle strenght, WOMAC, Merlè d'Aubignè-postel	THA control. Healthy control
Quagliarella 2010	81(nr-nr)-64.1-nr	retrospective	12-6 months	no	nr	platform	Healthy control
Rasch 2010	22(18-4)-67-nr	prospective	24-6 months	no	10weeks training post-op	muscle strength, balance (platf),gait	pre-post surgery
Bitterli 2011	39(19-22)-65.4-nr	prospective RCT	10day-4-12	no	4w pre-op sensorimotor training	SF-36,WOMAC,BBS (platf)	pre-post surgery. Control no training
Kiss 2012	80(47-33)-71.3-nr	prospective	2003/6/12	no	direct lateral or anterolateral, BMI	HHS, gait,	healthy control
Holnapy 2013	45(36-9)-nr-nr	prospective	6weeks -3-6	no	direct lateral or anterolateral or posterior	perturbation platform	pre-post surgery.-other approaches
Larkin 2013	100(38-62)-nr-nr	prospective	nr	nr	hip resurfacing and THA	UCLA score, Harris hip, dynamic postural stability	pre-post surgery, healthy matched control, different intervention

Brauner 2014	61(27-34)-61.8-nr	prospective	2weeks	no	2weeks rehabilitation post-op	interlimb vertical force and dynamic stab during squat	healthy control
Caplan 2014	14(1-14)-63.8-nr	prospective	12-3 months	no	hip resurfacing-THA	TUG, vertical ground reaction force	THA control. Healthy control
Jogi 2014	26(nr-nr)-64-nr	prospective RCT	1 1/2	no	5weeks training e balance train post-op	Berg Balance, TUG, WOMAC, ABC test	no training control
Butler 2015	75(nr-nr)-66.1-31	retrospective	12	yes	BMI	unilateral stand position	control joints replacement
Chang 2015	23(13-10)-60-nr	prospective	2w-6w-3-6	no	minimally invasive THA	Vas, Berg balance, FIM,A BC, foot placement	pre-post surgery
Jensen 2015	38(13-25)-55-27.9	prospective RCT	6-2 months	no	hip resurfacing-THA	gait deviation index	pre-post surgery. THA control. Leg healthy
Jogi 2015	63(41-22)-67-nr	prospective RCT	6weeks	no	5weeks training e balance train post-op	platform (ellipse area)	no training control
Zeng 2015	59(28-31)-64-26.7	prospective random	6-3 months	no	12weeks training post-op	WOMAC,6MWT,TUG,UPST, ROM	no training control
Jo 2016	31(11-20)-65.4-nr	retrospective	3	no	nr	Berg balance, JPS, QOL, sf-36. (Proprioception) muscle mass	pre-post surgery
Esposito 2017	30(nr-nr)-69-28	prospective randomized	3days	no	crutches set-up post-op	VAS, Oxford hip score, platform	set up group
Ninomiya 2017	58(48-10)-68-22	retrospective	120	no	nr	muscle strength, single limb stance time, MWS, fall rate, IPAQ	healthy control

Pop 2018	55(31-24)-56-nr	prospective	24-36	no	4weeks rehabilitation. Female	platform (stabilometric), romberg	healthy control
